# Variation in the Ovine Abomasal Lymph Node Transcriptome between Breeds Known to Differ in Resistance to the Gastrointestinal Nematode

**DOI:** 10.1371/journal.pone.0124823

**Published:** 2015-05-15

**Authors:** Albin M. Ahmed, Barbara Good, James P. Hanrahan, Paul McGettigan, John Browne, Orla M. Keane, Bojlul Bahar, Jai Mehta, Bryan Markey, Amanda Lohan, Torres Sweeney

**Affiliations:** 1 School of Veterinary Medicine, Veterinary Sciences Centre, University College Dublin, Belfield, Dublin 4, Ireland; 2 Teagasc Animal & Grassland Research and Innovation Centre, Athenry, Co. Galway, Ireland; 3 Teagasc Animal & Grassland Research and Innovation Centre, Grange, Co. Meath, Ireland; 4 UCD Conway Institute of Biomolecular and Biomedical Research, University College Dublin, Belfield, Dublin 4, Ireland; Centers for Disease Control and Prevention, UNITED STATES

## Abstract

Texel lambs are known to be more resistant to gastrointestinal nematode (GIN) infection than Suffolk lambs, with a greater ability to limit infection. The objectives of this study were to: 1) profile the whole transcriptome of abomasal lymph node tissue of GIN-free Texel and Suffolk lambs; 2) identify differentially expressed genes and characterize the immune-related biological pathways and networks associated with these genes. Abomasal lymph nodes were collected from Texel (n = 6) and Suffolk (n = 4) lambs aged 19 weeks that had been GIN-free since 6 weeks of age. Whole transcriptome profiling was performed using RNA-seq on the Illumina platform. At the time of conducting this study, a well annotated *Ovine* genome was not available and hence the sequence reads were aligned with the *Bovine* (UMD3.1) genome. Identification of differentially expressed genes was followed by pathway and network analysis. The Suffolk breed accounted for significantly more of the differentially expressed genes, (276 more highly expressed in Suffolk v 162 in Texel; P < 0.001). The four most significant differentially expressed pathways were all related to immunity and were classified as: *Role of Pattern Recognition Receptors in Recognition of Bacteria and Viruses*, *Activation of IRF by Cytosolic Pattern Recognition Receptors*, *Role of RIG-I-like Receptors in Antiviral Innate Immunity*, and *Interferon Signaling*. Of significance is the fact that all of these four pathways were more highly expressed in the Suffolk. These data suggest that in a GIN-free environment, Suffolk lambs have a more active immune profile relative to the Texel: this immune profile may contribute to the poorer efficiency of response to a GIN challenge in the Suffolk breed compared to the Texel breed.

## Introduction

Gastrointestinal nematode (GIN) infection of ruminants is a major economic and health concern, causing substantial loss to livestock producers worldwide. Increasing consumer concerns about drug residues in animal products and the emergence of anthelmintic-resistant nematode species have stimulated efforts to develop alternative strategies to anthelmintic therapy [1], including genetic selection for natural resistance to nematodes. The latter approach is justified by evidence of genetic variation in resistance to GIN infection both within and between sheep breeds [2–6].

The identification of genetically resistant animals through DNA analysis has been focused, to date, on the identification and characterization of candidate genes such as the major histocompatiblity complex (MHC) *DRB* [5, 7], MHC class I and II genes [8, 9] and interferon gamma [10, 11], all of which have alleles that have been associated with resistance to GIN. A weakness of the candidate gene approach is that resistance/susceptibility to GIN is a complex trait with genetic control being polygenic. Our hypothesis is that biochemical pathways and networks are central to differences in resistance and, thus, identification of these will provide a more secure foundation for the elucidation of gene profiles associated with resistance to GIN. Others have shown that a number of genes/biological processes are differentially expressed in duodenal tissue from two lines of lambs selected for divergence in resistance to GIN [12, 13].

Previous studies from our group have shown that the Texel breed is more resistant to GIN infection than the Suffolk breed [14]. Comparing the gastrointestinal lymph tissue transcriptome between Texel and Suffolk in the absence of a GIN challenge should allow the identification of gene pathways and networks that are differentially expressed between the two breeds and thus likely to be involved in the response to a GIN challenge. An understanding of the molecular basis for such breed variation may reveal markers associated with resistance to pathogens. Hence, the objectives of the present study were: 1) to profile the whole transcriptome of abomasal lymph node tissue of GIN-free Texel and Suffolk lambs; and 2) to identify the differentially expressed genes and characterize the immune-related biological pathways and networks associated with the difference in resistance between the Texel and Suffolk breeds. As a well annotated *Ovine* genome was not released at the time of conducting this study we aligned the transcriptome with that of the UMD3.1 *Bos taurus* genome.

## Materials and Methods

### Ethics statement

All procedures described in this experiment were conducted under experimental license (B100/2584) from the Irish Department of Health in accordance with the Cruelty to Animals Act 1876 and the European Communities (Amendments of the Cruelty to Animals Act 1976) Regulations, 2002 and 2005) and approved by the Teagasc Ethics Committee (5909).

### Animals

The lambs described here were part of a larger experiment concerning the response of Suffolk and Texel lambs to experimental infection with Teladorsagia circumcincta [15]. Lambs (10 in total; 6 Texel and 4 Suffolk) were born (28th and 29th March) indoors, and moved to pasture until 6 weeks of age (mid-May), at which point they were weaned and moved and kept indoors until mid-September. Given that *Nematodirus battus* was the predominant species observed in FEC in lambs after housing (all lambs, data not shown), it was the predominant species on pasture. Animals were maintained indoors on a concentrate-based diet, with free access to water, from mid-May to mid-September. The lambs were faecal sampled per rectum at weaning but sufficient material was obtained from only 7 individuals (4 Texel and 3 Suffolk). Nematodirus eggs were detected in all cases (17, 50, 150, 1700 e.p.g. for Texel and 22, 300, 1000 e.p.g. for Suffolk) while eggs from other Trichostrongyle spp (excluding *S*. *papillosus*) were detected in 4 cases (1, 17, 100 e.p.g. for Suffolk and 50 e.p.g. for one Texel). All lambs were then treated with Ivermectin (Oramec, Merial Animal Health Limited) according to the manufacturer’s instructions and quarantined in a slatted pen for 48 h prior to being penned on straw. Faecal samples were collected on 3 consecutive days 5 weeks post-housing/anthelmintic treatment to determine GIN infection status. No eggs were detected in the faecal material of any of the 10 lambs at this stage. The lambs were faecal sampled again one week prior to slaughter to confirm their trichostrongyle infection-free status for at least 13 weeks prior to slaughter.

### Tissue samples

At the end of the experiment, all ten animals were slaughter by electrical stunning followed by exsanguination. Immediately after slaughter, animals were dissected and approximately 1 g of abomasal lymph node was stored from each animal at room temperature for 24 h in 10 mL RNAlater (Ambion, Austin, TX, USA). RNAlater was then discarded and samples were stored at -80°C until RNA was isolated.

### RNA extraction

A representative subsample (40 to 50 mg) of the abomasal lymph node tissue was homogenized in 1 mL of TRIreagent (MRC, Cincinnati, OH, USA), using the Qiagen TissueLyzer, and total RNA was extracted following the manufacturer’s instructions. DNase I (Sigma-Aldrich, St Louis, MO, USA) treatment of the RNA was then performed and further purification was carried out using the GenElute mammalian total RNA miniprep kit (Sigma-Aldrich, St Louis, MO, USA). The quantity and quality of total RNA were assessed using a Nano Drop spectrophotometer (Thermo Fisher Scientific, USA) and an Agilent 2100 Bioanalyzer (Agilent Technologies, Inc. CA, USA), respectively. All RNA samples used in this study had an RNA integrity value ≥ 8.0.

### RNA-seq library preparation

An RNA-seq library was prepared from ~2 μg of total RNA using an Illumina TruSeq RNA Sample Prep Kit (Cat. No FC-122-1001; Illumina Inc., San Diego, CA, USA) according to the manufacturer’s protocol. A multiplexing-capable kit was used for sample multiplexing. The final concentration of the libraries was assessed using a Qubit fluorometer (Invitrogen, Paisley, UK) and quality was assessed using a high sensitivity DNA kit (Agilent Technologies, Inc. CA, USA). The high sensitivity DNA chip and the Agilent 2100 Bioanalyzer were used for library sizing and final validation. One RNA-seq library was prepared from abomasal lymph node tissue from each animal.

### Sequencing

Equi-molar quantities (10 nM) of the bar-coded cDNA libraries were multiplexed and run in 6 lanes across three flow cells. The sequencing was carried out using Illumina’s Genome Analyzer II (Illumina Inc., San Diego, CA, USA) according to the manufacturer’s instructions. The sequencing products consisted of single-end reads of 36 nucleotides plus the 6-nucleotide index marker (42 bases in total). FastQC [16] software was used to assess the quality of the sequence data; sequences with a Phred mapping quality score ≥ 30 were used for further analysis. This quality score threshold corresponds to base call accuracy of 99.9% [17]. As the quality of the sequence data was very good we did not trim the raw sequences during the processing of data, and thus the length of raw and processed reads were same.

### Data analysis

As a well annotated *Ovine* genome was not released at the time of conducting this study we aligned the transcriptome with that of *Bovine*. The alignment software Bowtie 0.12.7 [18] was used to align the reads to the UMD3.1 *Bos taurus* genome assembly allowing up to two mismatches per read. Only uniquely aligned reads were used for the analyses. The binary alignment/map (BAM) files from the Bowtie mapping were sorted and filtered for duplications (possibly resulting from PCR bias) using SAMtools [19]. The raw counts, per gene were estimated by HTseq (v0.4.7) (http://www-huber.embl.de/users/anders/HTSeq/doc/overview.html#). The data were normalized in edgeR [20] using the trimmed mean of M values (TMM) method [21]. Only genes with ≥ 5 reads in total, across all samples, were included in the final analysis. The normalized raw counts per gene were used to identify genes that were differentially expressed (DE) between breeds using edgeR. Gene ontology (GO) of DE genes was performed using GO-Elite [22]. Classification of functional processes of biological importance and of canonical pathways and networks of DE genes were performed using Ingenuity Systems Pathway Analysis (IPA; Ingenuity Systems, Redwood City, CA, USA; http://www.ingenuity.com). Data has been made publically available: http://www.ncbi.nlm.nih.gov/geo/query/acc.cgi?acc=GSE43241.

As some of the most significant differentially expressed pathways suggested that the animals may have been infected with a virus, the RNA-seq data were further aligned with the reference genomes of a number of different viral species [orf virus (HM133903.1) virus, jaagsiekte sheep retrovirus (NC_001494.1), Schmallenberg (NC_018459.1), bluetongue virus (NC_006023.1)]. Moreover, the findings of alignment (presence of orf virus) were further confirmed by using conventional polymerase chain reaction. Genomic DNA was prepared from 100 mg of abomasal lymph node tissue samples. Amplification of orf virus was carried out according to Inoshima *et al*. [23]. The forward- (5'GCGAGTCCGAGAAGAATACG3') and reverse (5'TACGTGGGAAGCGCCTCGCT3') primers bind to a target region specific to the major envelope protein (B2L) gene of orf virus isolate SV178/12 and yielding a PCR product of 594 bp. The reaction conditions and PCR cycling conditions are as described by Inoshima *et al*. [23].

### Validation by quantitative real time PCR (qPCR)

To validate the results of RNA-seq analysis, a panel of differentially expressed genes was selected from the RNA sequence database and qPCR assays were designed to confirm the direction and magnitude of the expression profiles. Ten genes were randomly selected from the panel of 27 DE genes identified and classified as part of the genetic network ‘*Infectious Disease*, *Antimicrobial Response*, *Inflammatory Response*’. Gene specific primers were designed using Primer Express (v2.0) (PE Applied Biosystems) software and were synthesized by Sigma Aldrich, USA. The details of the primers are in Table 1.

**Table 1:** Nucleotide sequence of primers used for quantitative real time PCR.

Gene category	Gene Symbol	Sequence (5^/^ to 3^/^)	Product (bp)
Reference genes	*PGK1*	TTGGCACTGCTCACCGAGCC TCGGGGCTCTCCAAGGCCTT	118
	*RPL19*	ATGCCAACTCCCGCCAGCAG CTTCCGGCGAGCCAAGGTGT	116
	*GAPDH*	ATGCCTCCTGCACCACCA AGTCCCTCCACGATGCCAA	76
	*B2M*	TTCTGTCCCACGCTGAGTTCA CAACCCAAATGAGGCATCGT	149
	*TBP*	GACCATTGCACTTCGTGCCCG CTCTTGGCTCCCGTGCACACC	135
	*ACTB*	CGCAGACAGGATGCAGAAAGA GCTGATCCACATCTGCTGGAA	148
	*GUSB*	CTCATCGTTGGTGCCAATGCAAGT TCACATCCACCCTGGGAAACAGAA	193
	*ATPSynth*	TCCTGCTCTGATCCGTTCTT GGCCACTGCTGTAGGAAGG	107
Target genes	*EPCAM*	AATGGTGAACTACTGGATCTG CCACAATGACGGCAATAATAC	115
	*IFIT1*	GAAGAAGCACTGACTGATGAG TGGATTATTTGTGACTTGTAGCA	182
	*ISG-54*	CAGAGACTAATAAGACACGCTAT TTCTTCATACTGACCGACTTG	120
	*IFIT3*	TCCATACCAAACAATGCCTAC AGTTCTTCAATCTCCTCTCCTT	122
	*IFIT5*	TCCAGAGATTGACTGTGAGAA TCAGAATCATCCAGCCGATA	162
	*MDA-5*	GTCGTCGGATGGGAGTTT GATGTACCTTTTCACTCTGGC	79
	*OAS1*	TCTTCCTCACCAATCTCACA GACCTCAAACTTCACTTCAAATG	125
	*RIG-I*	GATGAATGTCACAACACCAAT GCAATGAGTCTGAAGATCCT	94
	*RSAD2*	TAATCTGTAGCCCATACTGACTAT AACTCCATCACAAAGCGTAAA	106
	*MX1*	ATGGTTCTTTCTGACTTGGAT CCTTGGACTCCGTTTCAT	100

Complementary DNA (cDNA) was synthesized from 1 μg of purified total RNA using cDNA Synthesis Kit (Fermentas, Vilnius, Lithuania) and oligo dT primers following the manufacturer’s instructions. The primer efficiency was determined using a serial dilution (1:4 dilution series over 7 points) of a cDNA pool, prepared by pooling an equal quantity of cDNA from all of the experimental samples; the efficiency of all primers was shown to be between 90% to 110%. The reaction was carried out in a total volume of 20 μL, which comprised 10 μL 2X Fast SYBR green PCR Master Mix (Life Technologies), 1 μL of forward and reverse primer mix (final concentration 300 nM each), 1 μL of diluted cDNA template (equivalent to 2.5 ng of RNA) and 8 μL of nuclease-free water (Sigma-Aldrich, St Louis, MO, USA), in an ABI Prism 7500 FAST sequence detection system (Applied Biosystems, Warrington, UK). The PCR cycling conditions were 95°C for 10 min for 1 cycle followed by 95° C for 3 sec and 60° C for 30 sec for 40 cycles. The specificity of the product was confirmed by melt curve analysis.

Following analysis of the relative quantities of the gene transcripts of 8 potential reference genes (*GAPDH*, *B2M*, *PGK1*, *ATPsynth*, *RPL19*, *TBP*, *GUSB*, *ACTB*) with the GeNorm application within the qBase^PLUS^ software package [24] (Biogazelle, Zwijnaarde, Belgium), two robust reference genes (*PGK1* and *RPL19)* were selected for normalization of the relative expression of the target genes. Calibrated normalized relative quantities of gene expression for each target gene were generated using qBase^PLUS^. RNAseq and qPCR data were compared by calculating the correlation coefficient for specific genes after accounting for breed effects using SAS v9.1.3 (SAS Institute, Cary, NC, USA).

## Results and Discussion

An average of 13 (s.d. 1.9) million reads were generated per sample. Due to the lack of a well-annotated *Ovine* genome at the time of conducting this study, reads were aligned to the bovine sequence, which was the closest species with a well-annotated genome. Alignment of these reads to the *Bos taurus* genome yielded an average of 7 (s.d. 1.1) million uniquely aligned reads per sample, which were subsequently used in the analysis.

### Differentially expressed genes and validation of RNA-seq data

**Fig 1:**
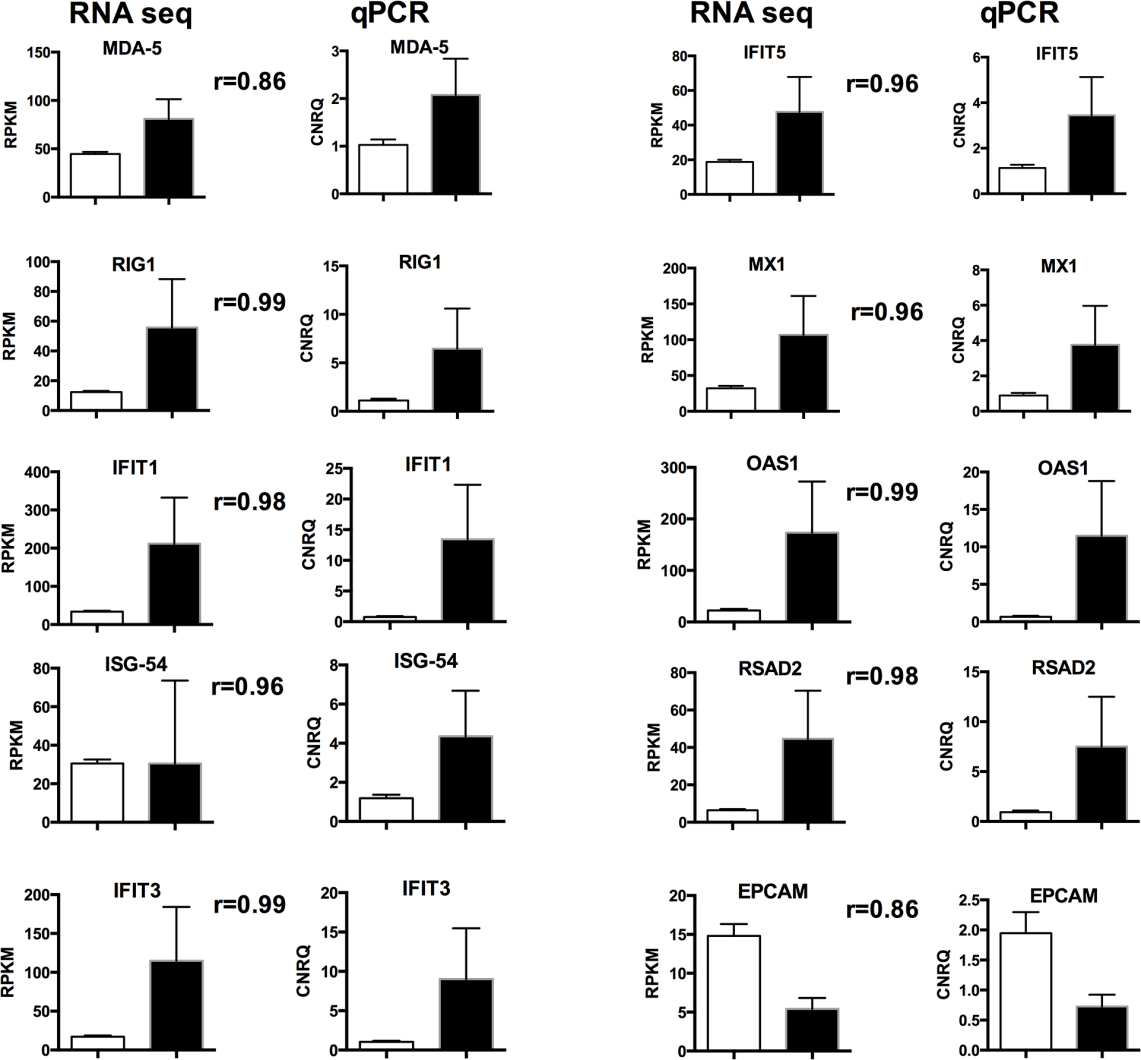
Validation of RNA-seq data using quantitative real time PCR. Clear bar shows the Texel breed and the black bar represents Suffolk breed. Error bars show the standard error of the mean. Pearson correlation coefficient (r) values within breed effect (P<0.001 for all comparisons) are shown between the RNA-seq and qPCR charts.

Based on a false discovery rate < 0.05, a total of 437 DE genes were identified. The gene expression patterns, in terms of direction and magnitude, of all 10 genes chosen for validation were reproducible by qPCR analysis (Fig 1). Of the DE genes, 276 were more highly expressed in Suffolk while 161 were more highly expressed in Texel. The proportion of DE genes was significantly associated with breed (P < 0.001).

### Gene-ontology analysis of differentially expressed genes

Functional annotation classification of the 276 genes more highly expressed in the Suffolk breed yielded a total of 59 significant (P< 0.05) functional annotation clusters; the 10 most significant clusters are presented in Table 2. The majority of the functional annotation clusters in the Suffolk relate to the immune system, including: *ISG15-protein conjugation*, *Immune response*, *Antigen processing and presentation of peptide antigen via MHC class I*, *Response to other organism*, *Defence response*, *MHC class I protein complex*, *Regulation of interleukin-10 production*, *Chemokine activity and Type I interferon biosynthetic process*. The most significant group, *ISG15-protein conjugation*, contains 4 genes, whereas the two largest clusters *Immune response* and *Defence response* comprise 31 and 21 genes, respectively. In the Texel breed, a total of 26 significant (P < 0.05) functional annotation clusters were identified and the 10 most significant clusters are presented in Table 3. The GO terms associated with highly expressed genes in the Texel breed describe normal cellular processes and include: *Vitamin transport*, *Regulation of smooth muscle cell proliferation*, *Collagen fibril organization*, *Proteinaceous extracellular matrix*, *Extrinsic to plasma membrane*, *Extracellular space*, *Glucose metabolic process and Cell-cell adhesion*.

**Table 2:** Major functional annotation clusters (top 10 annotation clusters based on z-score) of genes that were differentially expressed in Suffolk breed.

GO name	Number of genes	z-score	Genes names
ISG15\-protein conjugation	4	16.47	*USP18*, *UBA7*, *UB2L6*, *ISG15*
Immune response	31	9.59	*RIG-I*, *LAG3*, *C1QB*, *IL1A*, *TBK1*, *RSAD2*, *OAS2*, *OAS1*, *CXL10*, *CTLA-4*, *MHC-I*, *C1QC*, *TNFRSF4*, *CCL22*, *BOLA*, *A6QQE3*, *IDO1*, *ANXA3*, *MIC*, *CACB3*, *CCL20*, *CCL8 oas1z*, *CD40L*, *FCERG*, *JSP1*, *Q3YFG6*, *Q9TSB2*, *Q9TUD0*, *REG1*, *TNFSF10*
Plasma membrane organization	4	9.23	*AGRN*, *DYSF*, *MYOF*, *Q9TUD0*
Antigen processing and presentation of peptide antigen via MHC class I	4	8.84	*BOLA*, *FCERG*, *JSP1*, *Q3YFG6*
Response to other organism	15	7.55	*STAT4*, *RIG-I*, *MX1*, *MX2*, *IRF3*, *TBK1*, *RSAD2*, *ISG20*, *A6QQE3*, *IDO1*, *ANXA3*, *FCERG*, *LYSCN*, *Q9TUD0*, *UCRP*
Defence response	21	7.16	*RSAD2*, *TBK1*, *RIG-I*, *IFI47*, *IL1A*, *LAG3*, *C1QB*, *TNFRSF4*, *CCR4*, *A6QQE3*, *IDO1*, *ANXA3*, *CCL20*, *CCL8*, *CD40L*, *CXL10*, *FCERG*, *LYSCN*, *Q56J78*, *Q9TUD0*, *REG1*,
MHC class I protein complex	5	6.97	*BOLA*, *B2MWQ2*, *MHC-I*, *JSP1*, *Q3YFG6*
Cellular response to heat	3	6.52	*IL1A*, *MYOF*, *TFEC*
Regulation of interleukin\-10 production	3	6.25	*IDO1 FCERG*, *TRIB2*
Chemokine activity	5	6.04	*CCL22*, *CCL20*, *CCL8*, *CXL10*, *REG1*

**Table 3:** Major functional annotation clusters (top 10 annotation clusters based on z-score) of up-regulated genes in Texel breed.

GO name	Number of genes	z-score	Gene names
Vitamin transport	3	9.40	*RET4*, *SLC22A16*, *TCN2*
Regulation of smooth muscle cell proliferation	3	7.43	*CAD13*, *CALRL*, *IBP5*
Collagen fibril organization	3	6.78	*COBA1*, *DERM*, *TNXB*
Proteinaceous extracellular matrix	9	6.05	*TNC*, *COBA1*, *DERM*, *FMOD*, *MFAP5*, *TNXB*, *PGS2*, *GPC1*, *POSTN*
Extrinsic to plasma membrane	3	4.58	*GBG7*, *SCUBE1*, *ST14*
Extracellular space	11	4.16	*TNC*, *A2M*, *ECM1*, *ADIPO*, *CAD13*, *DERM*, *FMOD*, *TNXB*, *PGS2*, *RET4*, *SCUBE1*
Extracellular matrix part	4	3.97	*TNC*, *COBA1*, *MFAP5*, *TNXB*
Cell projection assembly	4	3.90	*KLF5*, *CAD13*, *PKHD1*, *VANGL2*
Glucose metabolic process	4	3.56	*ADIPO*, *DCXR*, *PGM1*, *RET4*
Cell\-cell adhesion	7	3.50	*CDH17*, *ADIPO*, *CAD13*, *CLD1*, *COBA1*, *TNXB*, *PKHD1*

### Identification of pathways and gene interaction networks

#### Pathway analysis

**Fig 2:**
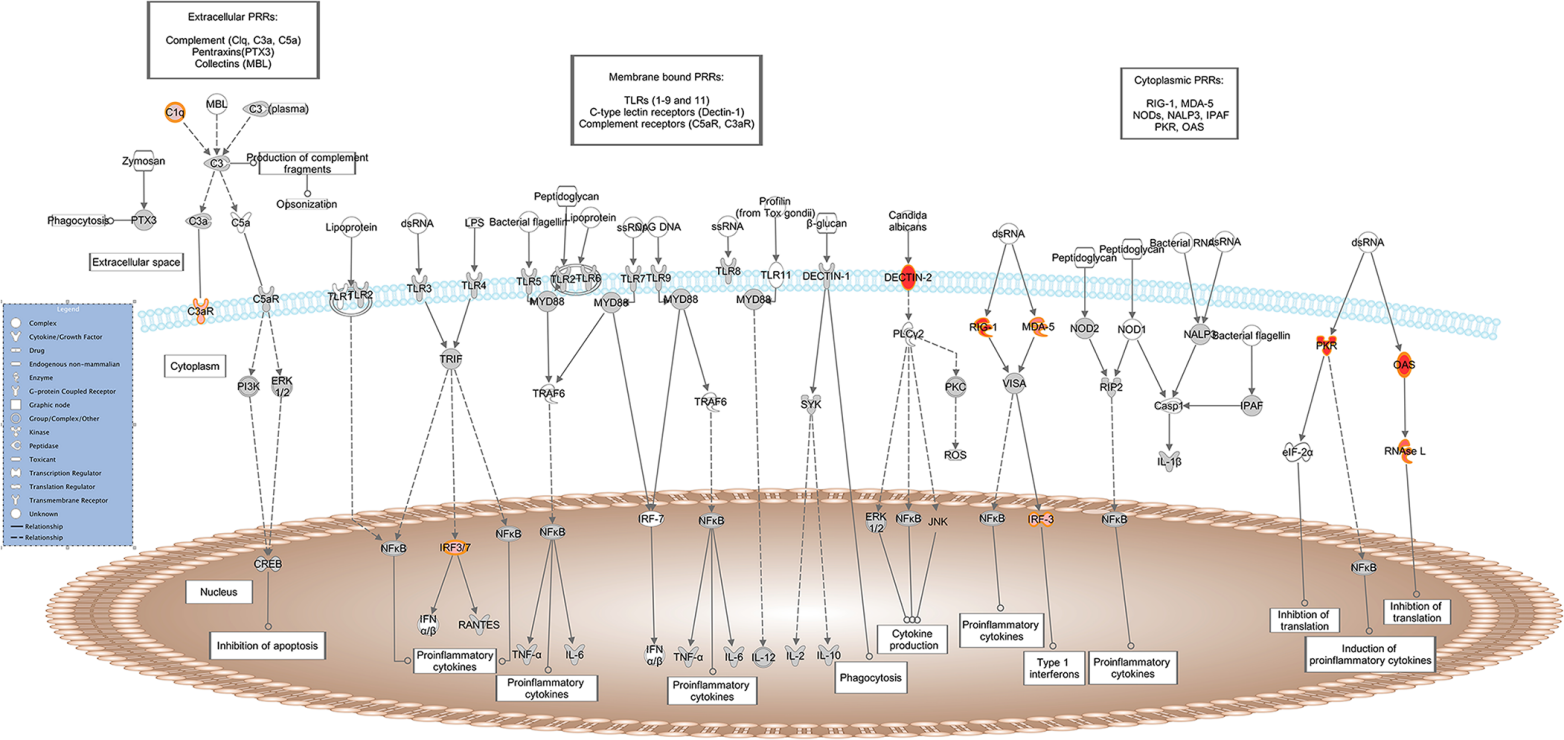
Schematic representation of the *Role of Pattern Recognition Receptors in Recognition of Bacteria and Viruses* pathway from IPA. Genes within the pathway showing differential expression are highlighted in colour. The colour intensity indicates the degree of elevated expression in Suffolk (red) or in Texel (green). Grey shading indicates genes that were not differentially expressed; white shading represents genes in the pathway not represented on the RNA-seq data.

**Fig 3:**
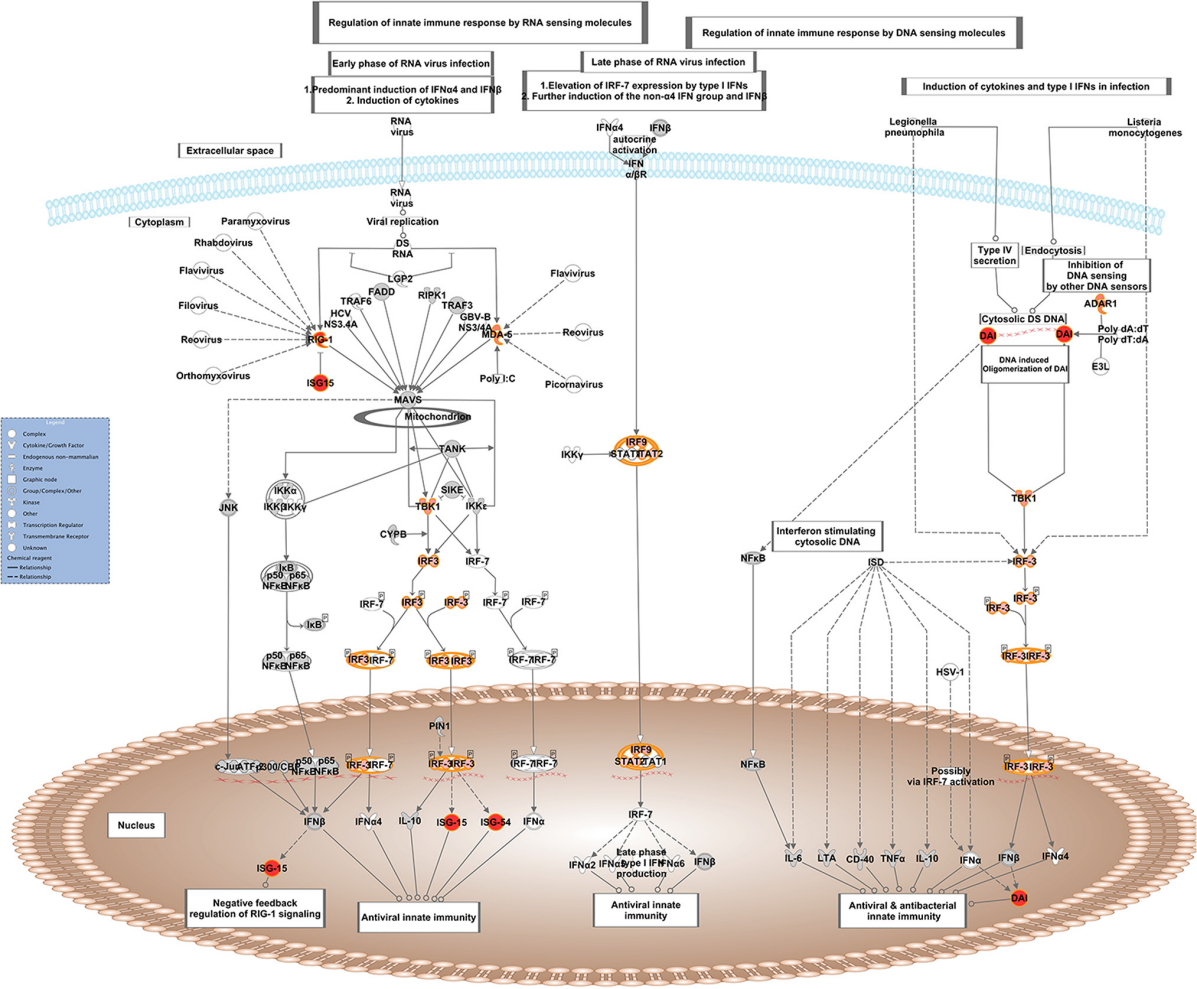
Schematic representation of the *Activation of IRF by Cytosolic Pattern Recognition Receptors* pathway from IPA. Genes within the pathway showing differential expression are highlighted in colour. The colour intensity indicates the degree of elevated expression in Suffolk (red) or in Texel (green). Grey shading indicates genes that were not differentially expressed; white shading represents genes in the pathway not represented on the RNA-seq data.

**Fig 4:**
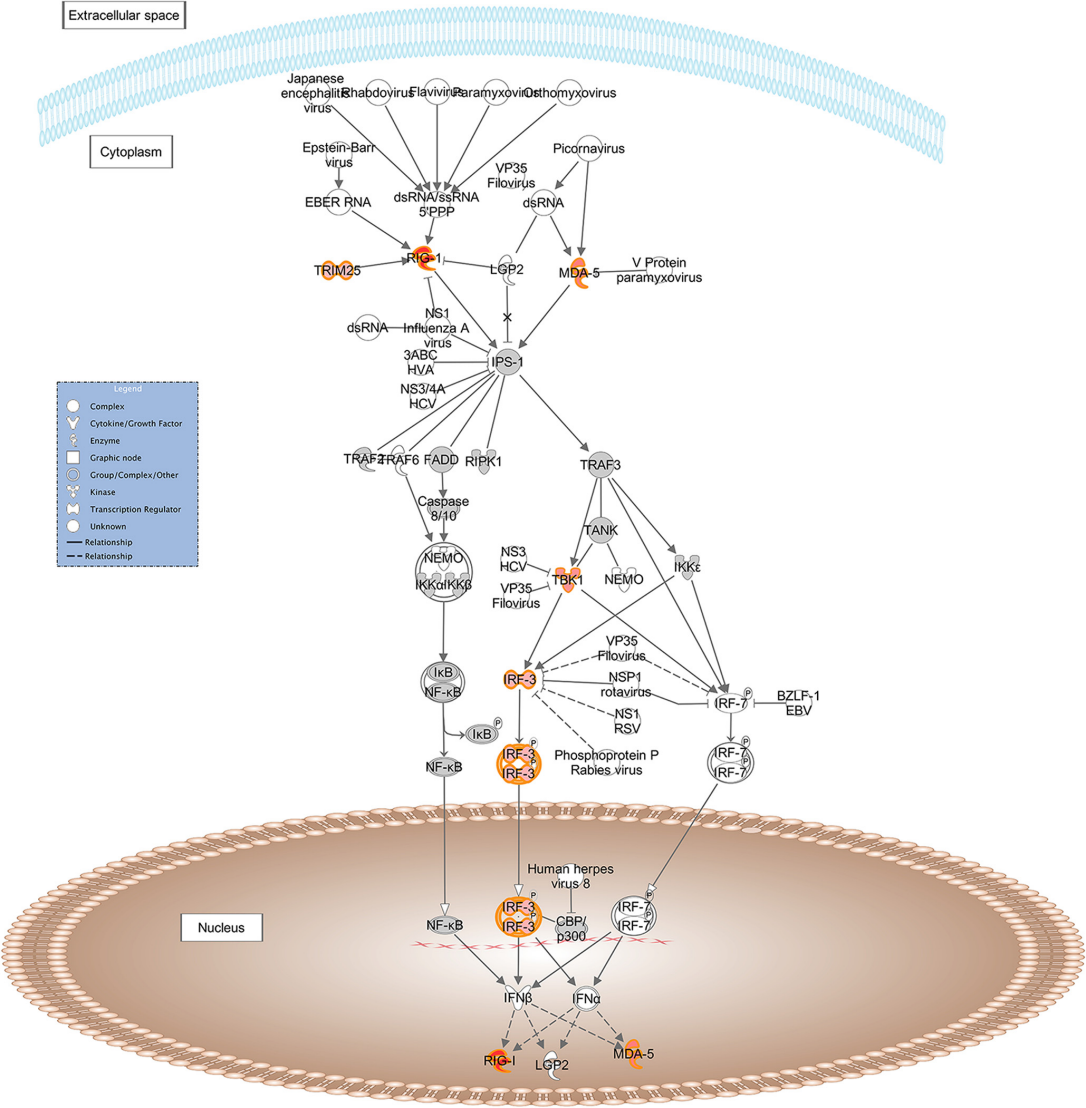
Schematic representation of the *Role of RIG-I likes receptors in antiviral innate immunity* pathway from IPA. Genes within the pathway showing differential expression are highlighted in colour. The colour intensity indicates the degree of elevated expression in Suffolk (red) or in Texel (green). Grey shading indicates genes that were not differentially expressed; white shading represents genes in the pathway not represented on the RNA-seq data.

**Fig 5:**
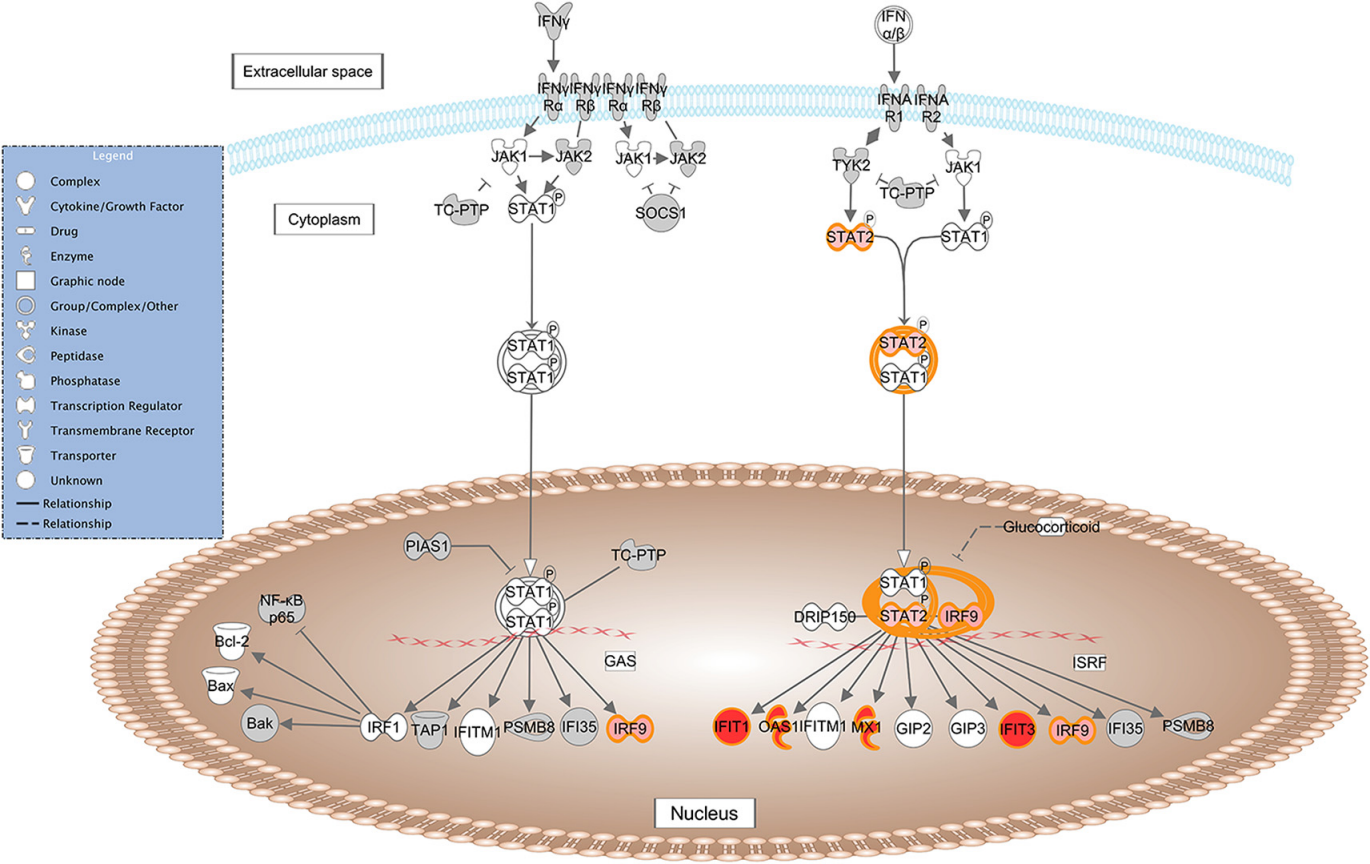
Schematic representation of the *Interferon Signaling* pathway from IPA. Genes within the pathway showing differential expression are highlighted in colour. The colour intensity indicates the degree of elevated expression in Suffolk (red) or in Texel (green). Grey shading indicates genes that were not differentially expressed; white shading represents genes in the pathway not represented on the RNA-seq data.

A total of 23 significantly over-represented canonical pathways were identified as differentially expressed between GIN-free Texel and Suffolk sheep (Table 4). The four most significant pathways all relate to processes of anti-viral /anti-bacterial innate immunity and were all more highly expressed in the Suffolk lambs relative to the Texel lambs. The four pathways and the DE genes assigned to each pathway are as follows:

- *Role of Pattern Recognition Receptors in Recognition of Bacteria and Viruses*: *RIG-I*, *MDA-5*, *DECTIN-2*, *IRF3*, *OAS1*, *OAS2*, *C1QC*, *C1QB*, *EIF2AK2*, *RNASEL*, *C3AR1* (Fig 2);
- *Activation of IRF by Cytosolic Pattern Recognition Receptors*: *RIG-I*, *MDA-5*, *DAI*, *ISG-54*, *TBK1*, *IRF3*, *IRF9*, *STAT2*, *ADAR1*, *ISG15* (Fig 3);
- *Role of RIG-I-like Receptors in Antiviral Innate Immunity*: *RIG-I*, *MDA-5*, *TBK1*, *IRF3*, *TRIM2*, (Fig 4);
- *Interferon Signaling*: *IFIT1*, *IFIT3*, *IRF9*, *OAS1*, *MX1*, *STAT*, (Fig 5).

**Table 4:** Gene classification based on canonical signaling pathways identified by Ingenuity pathway analysis (IPA).

Ingenuity Canonical Pathways	*P-values*	Molecules^‡^
Activation of IRF by Cytosolic Pattern Recognition Receptors	10.0×10^–08^	***MDA-5*, *RIG-I*, *DAI*, *STAT2*, *IRF9*, *TBK1*, *IRF3*, *ADAR1*, *ISG-54*, *ISG15***
Role of Pattern Recognition Receptors in Recognition of Bacteria and Viruses	7.0×10^–07^	***MDA-5*, *OAS1*, *OAS2*, *RIG-I*, *DECTIN-2*, *C1QC*, *C1QB*, *IRF3*, *EIF2AK2*, *RNASEL*, *C3AR1***
Interferon Signaling	2.1×10^–05^	***IFIT3*, *IFIT1*, *OAS1*, *MX1*, *STAT2*, *IRF9***
Role of RIG-I-like Receptors in Antiviral Innate Immunity	8.7×10^–04^	***MDA-5*, *RIG-I*, *TBK1*, *IRF3*, *TRIM25***
Riboflavin Metabolism	0.004	*FLAD1*, ***ENPP1*, *ACP6***
Communication between Innate and Adaptive Immune Cells	0.005	***CXCL10*,** *TLR10*, ***CD40LG*, *IL1A*, *HLA-A*, *FCER1G***
Dendritic Cell Maturation	0.006	***CD40LG*, *IL1A*, *HLA-A*,** *CD1A*, ***FCER1G*, *STAT2*,** *CREB3L4* **, *FCGR3A***
Graft-versus-Host Disease Signaling	0.008	***IL1A*, *HLA-A*, *FCER1G*, *GZMB***
Autoimmune Thyroid Disease Signaling	0.008	***CD40LG*, *HLA-A*, *FCER1G*, *GZMB***
Hepatic Fibrosis / Hepatic Stellate Cell Activation	0.010	***CD40LG*, *IL1A*, *CTGF*,** *MYL4*, *FGFR2*, *IGFBP5*, *A2M*
cAMP-mediated signaling	0.011	***P2RY13*,** *TULP2*, ***PRKAR2B*, *CCR4*, *PDE3B*, *ADCY6*,** *CREB3L4*, *CNGA3*, *MPPE1*
Sphingolipid Metabolism	0.012	***CERS6*,** *ARSJ*, ***SGPP1*,** *PPAP2C* **, *SMPD3***
G-Protein Coupled Receptor Signaling	0.014	*CALCRL*, ***GPR15*, *ADCY6*,** *CREB3L4*, *MPPE1*, ***ADRA1D*, *P2RY13*,** *TULP2*, ***PRKAR2B*, *CCR4*, *PDE3B*, *GPR126*, *CCR8*, *HRH4*, *C3AR1*,** *GPR143*
Altered T Cell and B Cell Signaling in Rheumatoid Arthritis	0.016	*TLR10*, ***CD40LG*, *IL1A*, *SPP1 (includes EG*:*20750)*,*FCER1G***
Complement System	0.018	***C1QC*, *C1QB*, *C3AR1***
Nitrogen Metabolism	0.023	*MARC1*, ***ADAR*,** *GLS2*
NF-κB Signaling	0.024	*TLR10*, ***CD40LG*, *IL1A*, *FCER1G*,** *FGFR2*, ***TBK1*, *EIF2AK2***
Cardiac β-adrenergic Signaling	0.255	*TULP2*, ***PRKAR2B*, *PDE3B*, *ADCY6*,** *GNG7*, *MPPE1*
Relaxin Signaling	0.026	*TULP2*, ***PRKAR2B*, *PDE3B*, *ADCY6*,** *GNG7*, *MPPE1*
Alanine and Aspartate Metabolism	0.028	*DDO*, ***AGXT2*,** *ASL*
Protein Kinase A Signaling	0.040	*TULP2*, ***PRKAR2B*, *ADD2*, *PDE3B*, *ADCY6*,** *MYL4*, *CREB3L4*, *CNGA3*, *GNG7*, *MPPE1*
VDR/RXR Activation	0.044	***CXCL10*, *SPP1 (includes EG*:*20750)*, *CYP24A1*,** *IGFBP5*
Lipid Antigen Presentation by CD1	0.049	*CD1A*, ***FCER1G***

‡Genes more highly expressed in the Suffolk breed are in bold while genes that are more highly expressed in the Texel breed are in normal typeface.

#### Gene interaction networks

**Fig 6:**
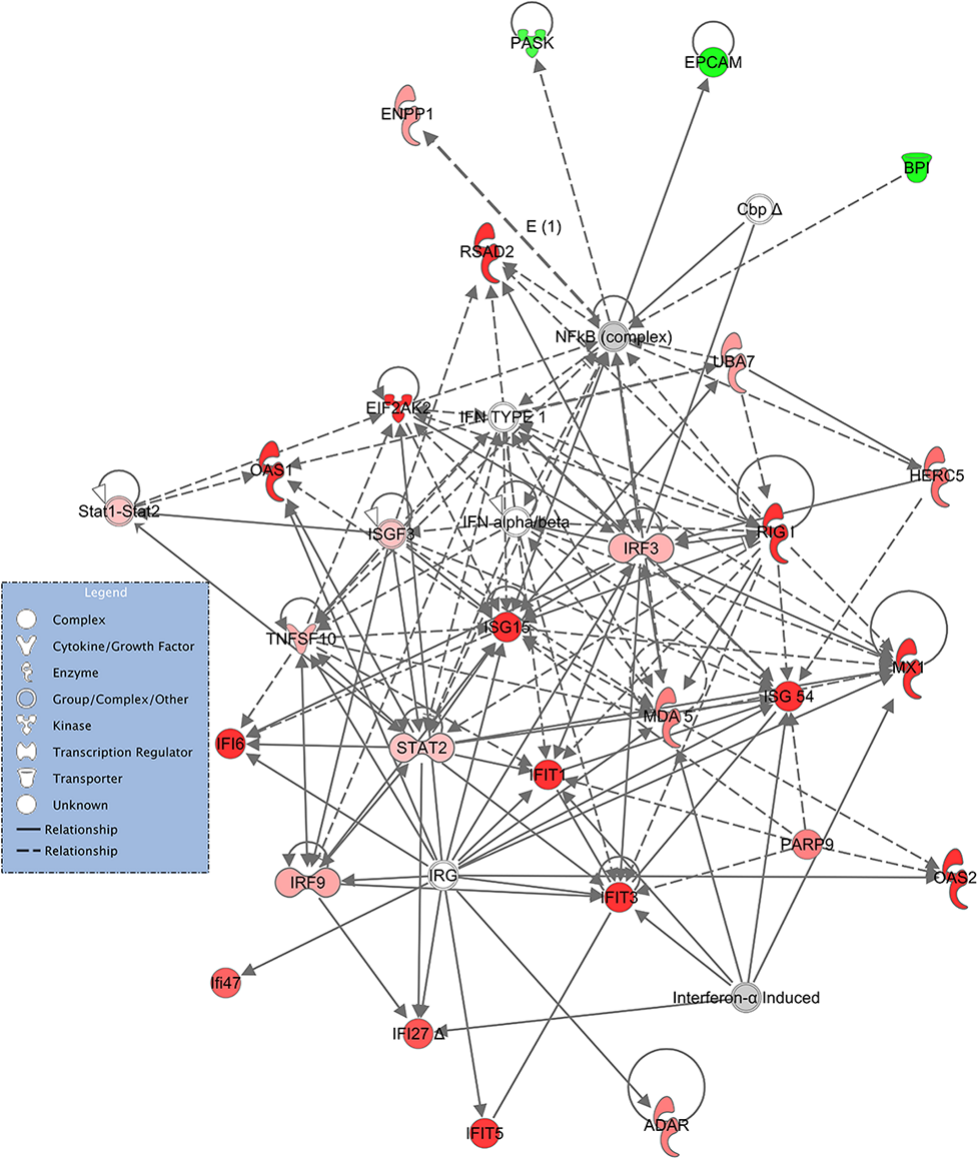
Schematic representation of the *Infectious Disease*, *Antimicrobial Response and Inflammatory Response* interaction network from IPA. Genes within the pathway showing differential expression are highlighted in colour. The colour intensity indicates the degree of elevated expression in Suffolk (red) or in Texel (green). Grey shading indicates genes that were not differentially expressed; white shading represents genes in the pathway not represented on the RNA-seq data.

The overall gene interaction networks for the differentially expressed genes were constructed using IPA. A total of 25 significant (P < 0.05) gene interaction networks were identified. The details of the 10 most significant networks are presented in Table 5. The gene interaction network with the highest number of focus molecules (n = 27) was designated *Infectious Disease*, *Antimicrobial Response*, *Inflammatory Response*. This network included 24 genes that were more highly expressed in the Suffolk breed and 3 genes that were more highly expressed in the Texel breed (Fig 6).

**Table 5:** Top 10 gene classifications of molecules in networks using IPA.

Top functions	Molecules^‡^ in Network	Score	Focus molecules
Infectious Disease, Antimicrobial Response, Inflammatory Response	***ADAR*,** *BPI*, ***RIG-I*, *EIF2AK2*, *ENPP1*,** *EPCAM*, ***HERC5*, *IFI6*, *IFI27*, *Ifi47*, *MDA-5*, *IFIT1*, *ISG-54*, *IFIT3*, *IFIT5*, *IRF3*, *IRF9*, *IRG*, *ISG15*, *MX1*, *OAS1*, *OAS2*,** *PASK*, ***RSAD2*, *STAT2*, *TNFSF10*, *UBA7***	40	27
Tissue Morphology, Cell-To-Cell Signaling and Interaction	***AGRN*, *C1QB*, *C1QC*,** *DCN*, *ELF5*, ***ETS2*, *EVI2A*,** *FMOD*, ***GBP7*, *GDF11*,** *GPC1*, *KLRC1*, ***KLRD1*, *LAG3*, *MHC Class I (complex)*, *MRPL44 (includes EG*:*301552)*,** *MYL4* **, *NAIP*,** *PPAP2C*, ***SGPP1*, *SIGLEC1***, *ST14*, ***TFEC*, *XAF1***	37	24
Connective Tissue Development and Function, Lipid Metabolism, Small Molecule Biochemistry	*CDH13*, *CDH17*, ***CDH20*,** *CIDEC*, ***CLEC1B*, *DECTIN-2*, *CTLA4*, *FCGR3A*, *ICOS*, *LY6E*,** *MPPE1*, ***PDE3B*,** *PLIN1*, ***PRKAR2B*, *SAA*, *SAMSN1*, *STMN2*,** *THRSP*, ***TRIM21*,** *TULP2*	29	20
Gene Expression, Gastrointestinal Disease, Inflammatory Disease	***BATF2*, *C1QB*,** *CDH13*, ***CLEC4A*, *CLEC4E*, *CLEC4F*, *CMPK2*, *DDIT4L*, *DTX3L*, *FAM26F*, *HERC6*, *IFI6*, *IL22RA2*,** *NAALADL1*, ***PARP9*, *SAMD9*, *SHISA2*,** *SLC22A16*, *TMC6*	27	19
Infectious Disease, Cell-To-Cell Signaling and Interaction, Cellular Movement	***ALDH9A1*, *APOL3*,** *BTN1A1*, ***CCL20*, *CCL22*,** *CLCA1*, ***CXCL10*, *IDO1*, *Oas*,** *PLB1 (includes EG*:*151056)*, *RNASE1*, ***RNASEL*, *TBK1*, *TNFRSF4*, *TRIM25*, *UBE2L6*, *USP18*, *DAI***	25	18
Small Molecule Biochemistry, Inflammatory Response, Drug Metabolism	*ADIPOQ*, ***BST2*, *C1RL*, *CD40LG*,** *FABP4*, ***GSTM1*, *Gstt3*, *HP*, *HPGDS*, *IL1A*, *LYZ*, *PLA2G2A*, *PLSCR1*, *SAA1*, *SERPINE2*, *SPINK5*, *UBD*,** *VANGL2*	25	18
Tissue Development, Connective Tissue Development and Function, Skeletal and Muscular System Development and Function	*ACVR1C*, ***ADCY6*, *CACNA1H*,** *CD1A*, ***CTGF*, *CYP24A1*,** *ECM1 (includes EG*:*100332249)*, *EPHA7*, ***FCER1G*,** *HOMER2*, *IGFBP5*, *KLF5*, *LY9*, ***P2RX7*,** *PAPSS2*, *SH3GL2*, ***SMPD3*, *SPP1 (includes EG*:*20750)*,** *TLR10*, *TNXB*	24	20
Cell-mediated Immune Response, Cellular Movement, Hematological System Development and Function	***ACP6*, *ADRA1D*, *ARRDC4*, *C3AR1*,** *C5orf13*, *CALCRL*, ***CCR4*, *CCR8*,** *GNG7*, ***GPR15*, *GPR126*,** *GPR143* **, *HRH4*, *P2RY13*, *RGS1*, *WARS***	20	16
Lipid Metabolism, Molecular Transport, Small Molecule Biochemistry	*ACRBP*, ***CERS6*,** *CIDEC*, *CLCA1*, ***D4S234E*,** *DDO*, ***HUNK*,** *KIRREL3*, ***LMO3*,** *PLIN4*, ***PODXL2*,** *POSTN*, *SCUBE1*, ***SLC41A2*, *TDRD7***	19	15
Cell-To-Cell Signaling and Interaction, Nervous System Development and Function, Behaviour	***ANXA3*,** *ASL*, ***BCO2*,** *DENND2A*, ***FADS3*,** *FMOD*, *GLS2*, ***HUNK*, *IGSF6*, *KLHDC8B*, *LYPD1*, *LYSMD2*, *RTP4*,** *STS*, ***TRIB2***	19	15

^‡^Genes more highly expressed in the Suffolk breed are in bold while gene that are more highly expressed s in the Texel breed are in normal typeface.

Exploring the roles of these specific genes, pathways and the network in more detail, it is evident that in a GIN-free environment, the Suffolk lambs had greater expression of a number of cytosolic pattern recognition receptors, membrane bound receptors, transcription factors and IFN-induced proteins than Texel lambs. Interestingly, a number of DE genes are assigned to more than one of these pathways (e.g., *RIG-I*, *MDA-5*, *STAT2*, *IRF-3*, *IRF-9*, *OAS1*), indicating that there is overlap between the pathways. These molecules are normally associated with the T helper cell type 1 (Th1) response to viral and/or bacterial exposure [25–28]. A variety of pattern recognition receptors (PRRs) were more highly expressed in the Suffolk lambs relative to the Texel lambs. PRRs are a primitive component of the innate immune system. They recognise microbe-specific molecules (pathogen associated molecular patterns; PAMPS), including viral RNA/DNA, lipopolysaccaride, mannose, bacterial peptides, peptidoglycans, lipoproteins and fungal glucans [26–29]. They can be located intracellularly, extracellularly, or membrane bound. The gene expression of three cytosolic PRRs (RIG-I, MDA-5 and DAI) was higher in the Suffolk lambs than in Texel lambs. Both RIG-I and MDA-5 belong to the RIG-1-like receptor family, whose members act as sensors of viral RNA. As represented in Fig 3, upon binding to viral RNA, these receptors induce type-1 interferon (IFN) production via serine/threonine-protein kinase (TBK1), interferon regulatory factor-3 (IRF-3) and ISG-15 [26]. The PRR DAI binds to microbial dsDNA, which induces type-1 interferon production via TBK1 and IRF-3 [27]. Hence, activation of any of these three PRRs leads to the production of IFN, which in turn activates a cluster of IFN-induced proteins, including a number of those genes that were more highly expressed in the Suffolk lambs: *IFIT1*, *IFIT3* and *MX1* which are activated via the transcription factors STAT2 and IRF9 [28, 30, 31]. It has previously been reported that the IRF9 protein binds with STAT2 protein to produce a set of IFN-induced proteins IFIT3, IFIT1, OAS1, OAS2 and MX1 to activate non-specific anti-viral immunity [32, 33]. In fact polymorphisms in the *OAS1* gene are associated with host susceptibility to various diseases [34, 35] and with resistance to viral infections [36].

The genes coding for two membrane-bound PRRs (DECTIN-2 and C3AR1) were also more highly expressed in the Suffolk. DECTIN-2 is a PRR that stimulates non-specific antifungal immunity [37, 38] and upon activation mediates a Th2 immune response [39]. There was, however, no evidence that the downstream effector molecules of DECTIN-2 (ERK, NFkB or JNK) were differentially expressed between the breeds (Table 4). C3AR1 is a receptor of the complement cascade [40, 41], and interestingly, C1QB and C1QC, which are the B and C chains of the first component of the classical complement pathway were more highly expressed in the Suffolk lambs.

**Fig 7:**
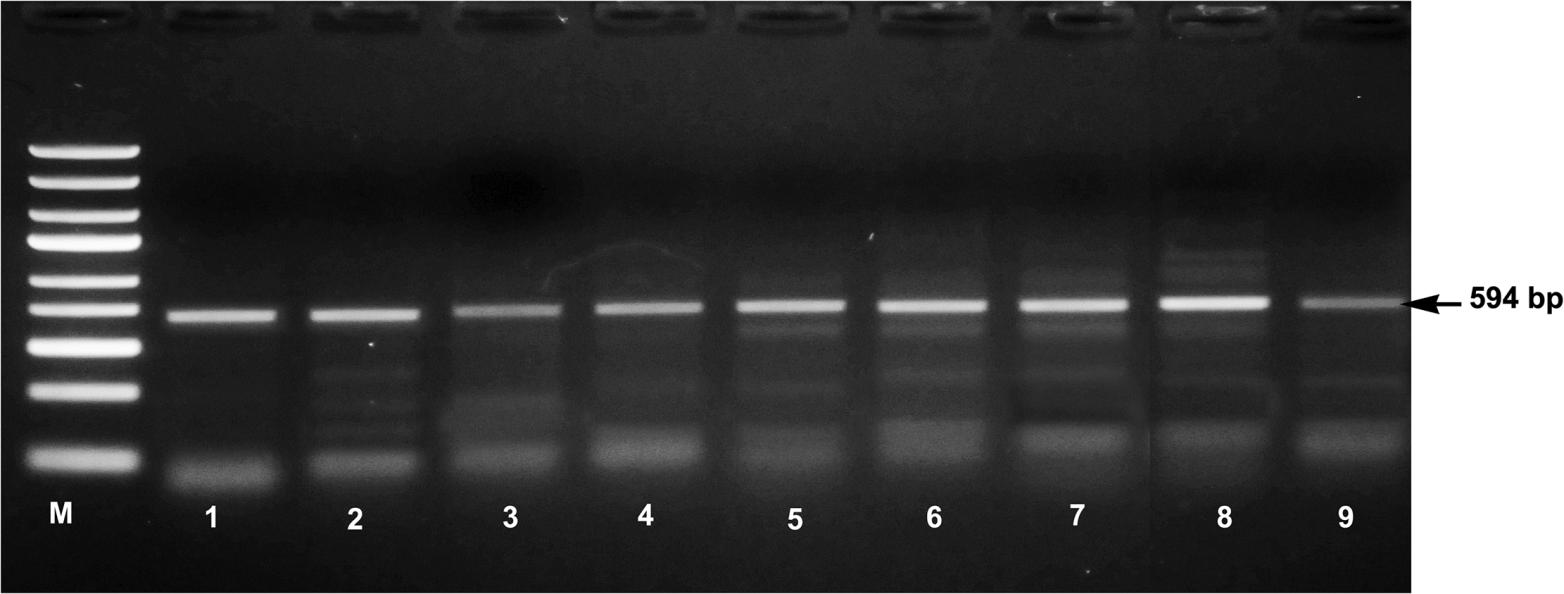
Polymerase chain reaction amplification of the 594 base pair region of major envelop protein (B2L) gene of orf virus isolate SV178/12 in Suffolk (Lane 1 to 4) and Texel (Lane 5 to 9) animals. M- molecular weight marker.

The spectrum of genes in the Th1/IFN network that are being transcribed in the Suffolk breed, suggests that the Suffolk animals are either innately more prepared for a microbial challenge than the Texel animals, or were actually actively responding to a microbial challenge. In exploring the possibility of recent microbial challenge, two approaches were adopted. Firstly, the haematological parameters of both Suffolk and Texel animals were assessed, and secondly, the RNA-seq data was searched for evidence of viral RNA from viral species known to infect sheep, including Schmallenberg virus, bluetongue virus, orf virus and jaagsiekte sheep retrovirus (JSRV). There was no evidence for an active infection based on the haematological parameters evaluated (unpublished data). Interestingly, there was evidence for two types of viral RNA in all the animals with a similar average number of reads in both breeds (JSRV: Suffolk 62±34, Texel 112±32; orf virus: Suffolk 2040±121, Texel 2190±152). It has previously been reported that the exogenous infectious form of JSRV has an endogenous counterpart, which was integrated into the ovine genome prior to the evolution of sheep and goats [42]. The sheep genome has approximately 27 integrated copies of endogenous JSRV that are genomically closely related to JSRV [43]. It is possible that the Suffolk lambs are responding to the products from the integrated JSRV genome and reacting in a manner described for autoimmune diseases [44]. However, the high number of reads aligned with the orf virus genome is highly suggestive of recent or active orf infection. Orf virus specific polymerase chain reaction in the sheep genomic DNA samples from the lymph node showed the presence of orf virus in all the animals (Fig 7). Viral DNA may be integrated with the genome of the animals and carried over within the cell during the life of the animals All of the animals used in this study received a fully virulent live virus vaccine (Scabivax, Intervet Ireland) during the first week of life. A recent study detected orf viral DNA in reindeer lymph nodes 4 weeks post experimental inoculation [45]. Within the confines of the current experiment, it was not possible to determine if either of these viruses are responsible for the immune profile in the Suffolk lambs. However, it is worth considering in our future studies particularly the alignment of this sequence data with *Ovine* genome are likely to reveal greater details on the nature of adaptive and innate immune system of these two breeds.

In conclusion, the analysis of the RNA transcriptome of Suffolk and Texel lambs maintained under GIN-free conditions, suggests that Suffolk sheep have a more active antiviral/antibacterial immune profile than Texels. It is likely that this profile contributes to the variation between the two breeds in their capacity to mount a timely and effective immune response when exposed to a gastrointestinal nematode challenge.
